# Passive heating following the prematch warm‐up in soccer: examining the time‐course of changes in muscle temperature and contractile function

**DOI:** 10.14814/phy2.12635

**Published:** 2015-12-03

**Authors:** Paul W. M. Marshall, Rebecca Cross, Ric Lovell

**Affiliations:** ^1^ Human Performance LaboratorySchool of Science and HealthWestern Sydney UniversitySydneyNew South WalesAustralia

**Keywords:** Muscle contractile function, muscular power, rate of torque development, voluntary activation

## Abstract

This study examined changes in muscle temperature, electrically evoked muscle contractile properties, and voluntary power before and after a soccer specific active warm‐up and subsequent rest period. Ten amateur soccer players performed two experimental sessions that involved performance of a modified FIFA 11+ soccer specific warm‐up, followed by a 12.5‐min rest period where participants were required to wear either normal clothing or a passive electrical heating garment was applied to the upper thigh muscles. Assessments around the warm‐up and cool‐down included measures of maximal torque, rate of torque development, muscle temperature (*T*_m_), and electrically evoked measures of quadriceps contractile function. *T*_m_ was increased after the warm‐up by 3.2 ± 0.7°C (*P* < 0.001). Voluntary and evoked rates of torque development increased after the warm‐up between 20% and 30% (*P* < 0.05), despite declines in both maximal voluntary torque and voluntary activation (*P* < 0.05). Application of a passive heating garment in the cool‐down period after the warm‐up did not effect variables measured. While *T*_m_ was reduced by 1.4 ± 0.4°C after the rest period (*P* < 0.001), this value was still higher than pre warm‐up levels. Voluntary and evoked rate of torque development remained elevated from pre warm‐up levels at the end of the cool‐down (*P* < 0.05). The soccer specific warm‐up elevated muscle temperature by 3.2°C and was associated with concomitant increases of between 20% and 30% in voluntary rate of torque development, which seems explained by elevations in rate‐dependent measures of intrinsic muscle contractile function. Application of a passive heating garment did not attenuate declines in muscle temperature during a 12.5‐min rest period.

## Introduction

Muscular power is the product of tension and velocity, and is one of the most important performance characteristics of humans in sports. For example, many sporting movements such as throwing, kicking, or striking occur in time periods less than 200 msec, which precludes the development of maximal torque. Mechanisms of power output are generally considered from peripheral factors within muscle (e.g., fiber type [Harridge et al. 1996], cross‐sectional area [Aagaard 2003], viscoelastic properties [Wilkie 1949; Blazevich et al. 2009]), and factors associated with providing central motor output to muscle (e.g., supraspinal drive [Johnson et al. 2015], motor unit firing rate [Van Cutsem et al. 1998], *α*‐motoneuron input‐output responses [Aagaard et al. 2002; Johnson et al. 2015]). Of particular interest within sporting contexts is the relationship between muscle temperature (*T*_m_) and power because increased *T*_m_ improves performance in explosive tasks such as vertical jumping and maximal sprint cycling (Bergh and Ekblom 1979; Sargeant 1987; Gray et al. 2006). Increased tension with temperature is caused by the attached myosin heads generating more force (Offer and Ranatunga 2015), mediated in part by elevated rates of ATP hydrolysis (He et al. 2000; Gray et al. 2006), with the net outcomes of improved amplitude and particularly rate‐dependent measures of muscle contractile function (Bottinelli et al. 1996; De Ruiter et al. 1999). Thus, increased *T*_m_ is a sought after outcome in sports, particularly prior to the sporting activity commencing, to enhance muscle contractile function and increase muscular power.

The manipulation of *T*_m_ prior to sport is often achieved via an active warm‐up that includes a range of activity specific movements across a variety of intensity levels (Bishop 2003). A 15‐min active warm‐ups have been observed to increase *T*_m_ between 2 and 4°C measured at depths from 2 to 4 cm (Bishop 2003; Faulkner et al. 2013a,b), with concomitant increases in muscular power of between 4% and 10% per 1°C increase in *T*_m_ (Bergh and Ekblom 1979; Sargeant 1987; Gray et al. 2006; Faulkner et al. 2013a,b). Some evidence exists to support the mechanistic rationale that elevations of *T*_m_ between 2 and 4°C will increase rate‐dependent measures of muscle contractile function, thus contributing to higher voluntary muscular power.

De Ruiter (De Ruiter et al. 1999) showed that electrically stimulated rate of force development of the human adductor pollicis muscle was influenced by muscle temperature, with higher temperatures associated with greater rate of force development. However, comparisons were only performed at 22, 25, 31, and 37°C (De Ruiter et al. 1999), which does not encompass the normal physiological range for *T*_m_ in a sporting context. Two subsequent papers provided evidence for the ability of a warm‐up to enhance muscle contractile function, although no direct measures of *T*_m_ were performed. Skof (Skof and Strojnik 2007) and Girard (Girard et al. 2009) observed shorter time to peak and ½ relaxation times for evoked twitch responses from the quadriceps. Moreover, both papers observed increased central motor output to the quadriceps that may also contribute to enhanced voluntary muscular power following warm‐ups (Skof and Strojnik 2007; Girard et al. 2009). As noted by the authors (Girard et al. 2009), the contribution of elevated *T*_m_ to the changes in both muscle contractile function and central motor output could not be determined. Thus, there is a gap in understanding whether the expected elevation of *T*_m_ following an active warm‐up contributes to both increased muscle contractile function and central motor output.

While the warm‐up is a preparatory tool prior to sporting events, it is also common for athletes to experience delays between completion of the warm‐up and the start of the event. For example, in the English Premier League post warm‐up rest periods typically average ~12.5 min (Towlson et al. 2013). *T*_m_ declines immediately post exercise (Saltin et al. 1970; Kenny et al. 2003) in an exponential fashion (Faulkner et al. 2013b), with concomitant declines in measures of muscular power (Faulkner et al. 2013a,b). Recent studies have reported that use of a passive heating garment applied to the legs for 30 min following an active warm‐up can attenuate declines in vastus lateralis *T*_m_ and knee extensor muscular power (Faulkner et al. 2013a,b). While promising, these studies did not provide mechanistic evidence regarding changes in in vivo muscle contractile function or central motor output that might explain the observed results. Moreover, the time period of 30 min exceeds the average post warm‐up rest period observed in a sport such as soccer. Indeed, inspection of the results suggest that differences in *T*_m_ between the passive heating garment and control condition measured at a depth of 2 cm only became apparent after 28 min of use (20 min if the heating garment was worn throughout the warm‐up [Faulkner et al. 2013b]). Therefore, it is unclear whether application of a passive heating garment for a 12.5‐min period commensurate with the average time between the end of the active warm‐up and start of professional soccer match‐play will be sufficient to attenuate declines in both *T*_m_ and measures of muscular power.

Therefore, we designed this study to address gaps in the understanding of the relationship between the manipulation of *T*_m_, in vivo measures of muscle contractile function, central motor output, and voluntary rate of torque development around an active warm‐up and subsequent rest period. Moreover, we examined the effect of a passive heating garment applied during a 12.5‐min rest period after the warm‐up. It was hypothesized that (1) the active warm‐up would increase *T*_m_ and voluntary rate of torque development, with concomitant increases in rate‐dependent measures of in vivo muscle contractile function and central motor output; and (2) the 12.5‐min time period would hinder the effectiveness of the passive heating garment in the rest period with similar declines in *T*_m_, voluntary and electrically stimulated rate of torque, and central motor output compared to the control condition,

## Materials and Methods

### Participants

Ten male amateur level competitive soccer players (mean ± SD: age, 20.0 ± 3.0 years; height 1.76 ± 0.06 m; weight 74.7 ± 4.2 kg) volunteered for this study. All participants gave written informed consent before the commencement of the study after all experimental procedures and associated risks had been explained. The sample size was estimated using the calculated effect sizes from previous work which documented changes in concentric quadriceps peak torque and *T*_m_ during a passive 15 min half‐time rest period in a simulated soccer match (Lovell et al. 2013). The effect sizes for reductions in both peak torque and *T*_m_ were *d *=* *1.0, thus the estimated sample size for this study based on an 80% power calculation and *α *= 0.05 was *n* = 10.

Testing was performed in the latter ¼ of the 2014 competitive season, and all players took part in at least one team training session in addition to a competitive match every week, as well as regular fitness and strength training. All players had the aforementioned training history for a minimum of 3 years. The study was approved by the Western Sydney University Human Research Ethics Committee. All procedures conformed to the Declaration of Helsinki. Participants were asked to avoid vigorous exercise for 24 h, caffeine for 12 h, and food for 2 h before every trial.

### Experimental design

All participants attended the laboratory for three sessions, with each session separated between 5 and 7 days. At the first session, participants were familiarized with all experimental procedures including performance of maximal voluntary contractions of the knee extensors with and without femoral nerve stimulation, intramuscular temperature measurement, and performance of the standardized warm‐up (see *Warm‐up protocol*). The order of the two subsequent experimental sessions (Heat or Con) were randomized among participants and performed at the same time of day. Diet and hydration strategies prior to and during the first experimental session (including ad libitum water intake) were recorded and replicated.

### Experimental trials

Upon arrival to the temperature controlled laboratory (21.9 ± 1.5°C; 53 ± 8% relative humidity), participants were seated in an isokinetic dynamometer (KinCom 125, Version 5.32, Chattanooga) while electromyography (EMG) and femoral nerve stimulation preparation procedures were completed (see *Neuromuscular assessment*). Skin temperature (*T*_S_) thermistors (ADInstruments MLT422/A, ML309 Thermistor pod) were then applied to both the quadriceps and an initial vastus lateralis (VL) muscle temperature measurement (*T*_m_) was performed on the right leg, followed by a series of submaximal isometric knee extensions at increasing intensity (two each at 25%, 50% and 75% of perceived maximum effort). Subjects then performed four 3‐ to 4 sec maximal isometric knee extensor contractions separated by 120 sec. Subjects were instructed to contract “as fast and forcefully as possible” with strong verbal encouragement provided throughout all maximal trials.

Participants then performed a standardized soccer specific warm‐up before resting for 12.5 min to replicate the common prematch routines of professional soccer players (Towlson et al. 2013). Heart rate (HR) was monitored via a wireless Polar heart rate (Polar Team System, Kempele, Finland) monitor for the duration of the warm‐up (average HR: 157 ± 16 beats min^−1^; maximum HR: 181 ± 15 beats min^−1^). Immediately following the standardized soccer specific warm‐up (average duration 17:37 ± 00:45 min), *T*_m_ was recorded, and a maximal knee extensor trial performed for 3 min following completion of the warm‐up.

During the post warm‐up rest period (timed from completion of the post warm‐up maximal trial) participants remained seated for 12.5 min while wearing either a passive heating garment on the lower limb (Heat) or standard sporting shorts (Con). The passive heating garment applied in the Heat condition included battery powered heating elements designed to cover the gluteus maximus, vastus medialis, rectus femoris, vastus lateralis, biceps femoris, semitendinosus and semimembranosus of both legs. There is no coverage over the adductor muscles to allow for variation in leg size between participants. The heating elements were capable of reaching 40–42°C, powered by a 14.8 V battery that generated 7.5 W to each heating pad. After 12.5 min, the passive heating garment was removed and all EMG leads were attached. *T*_m_ was measured immediately followed by a final maximal knee extensor trial (completed ≤40 sec after garment removal). *T*_S_ was recorded for the duration of the protocol with exception of during the sports specific warm‐up.

### Warm‐up protocol

The three sections to the warm‐up were (1) slow speed running exercises; (2) dynamic stretches; and (3) moderate pace running exercises with directional changes (Table 1). Sections one and three were adapted from the FIFA 11+ injury prevention scheme, which is an effective warm‐up routine developed to enhance acute power production and reduce injury occurrence in soccer (Junge et al. 2011; Bizzini et al. 2013; Impellizzeri et al. 2013). Typically the FIFA 11+ replaces section two of this warm‐up with a range of trunk and lower limb strengthening exercises. We chose to replace the strengthening exercises with dynamic stretching because of concerns which our research group has over the use of high‐intensity eccentric exercise of the former within a warm‐up (Marshall et al. 2015), in addition to performance benefits associated with the latter (Gelen 2010).

**Table 1:** Modified FIFA 11+ warm‐up routine administered. Further details regarding the actions administered in Parts 1 and 3 of this warm‐up are available from the f‐marc web‐site (www.f-marc.com/11plus)

Work Interval			Relief interval		Total distance (m)
Action	Distance (m)	Repetitions	Action	Distance (m)	
Part 1: Running Exercises					
Straight line running	20	6	–	–	120
Running – Hip Out	10	6	Light Jog	10	120
Running – Hip In	10	6	Light Jog	10	120
Running – Circling Partner	20	6	Light Jog	20	240
Quick Running – Forwards & Backwards	10	6	Light Jog	10	120
Part 2: Dynamic Flexibility					
Heel (“Butt”) kicks	10	2	Walk	20	40
High knees (running)	10	2	Walk	20	40
Alternate Heel touches (running)	10	2	Walk	20	40
Walking lunges	10	2	Walk	20	40
High knee pull (“hug”)	10	2	Walk	20	40
Leg swings (flexion and extension)	10	2	Walk	20	40
Part 3: Running Exercises					
Running across the pitch (75–80% maximum pace)	20	4	Jog	20	160
Running – Bounding	20	3	Jog	20	120
Running – Plant & Cut	20	3	Jog	20	120

### Intramuscular temperature

Muscle temperature (*T*_m_) was recorded before (PRE) and after the warm‐up (POST), and after the passive rest period (REST) while participants were seated in the dynamometer. A needle thermistor probe (MKA‐08050‐A, ELLAB, Copenhagen, Denmark) was inserted 4 cm perpendicular to the VL. To ensure consistency of measurement, the midpoint between the lateral epicondyle and the greater trochanter of the femur was marked. The needle remained in the muscle for 3 sec before *T*_m_ was recorded_,_ and was immediately removed.

### Neuromuscular assessment

Maximal isometric knee extension contractions were completed on the isokinetic dynamometer with the knee angle fixed at 90° of flexion (0° corresponding to full knee extension). Participants sat upright in the dynamometer with hip and knee flexion at 90°, and were securely strapped into the chair by an over the shoulder strap and a belt across both thighs. The center of rotation of the dynamometer lever arm was aligned with the femoral condyle of the knee. The lower leg was firmly strapped to the dynamometer, approximately 2 cm superior to the lateral malleolus. All torque signals were continuously sampled at 2000 Hz, and a 10 Hz digital low‐pass filtered was applied (Powerlab, ADI Instruments, Sydney, Australia).

Surface electromyograms from the vastus lateralis (VL) and vastus medialis (VM) muscles were recorded via pairs of Ag/AgCl surface electrodes (Maxensor, MediMax Global, Sydney, Australia). Prior to electrode placement, careful skin preparation was performed including removal of excess hair, abrasion with fine sand paper and cleaning the area with isopropyl alcohol swabs. The VM electrodes (10 mm diameter, 10 mm interelectrode distance) were placed 2–3 cm above the superior portion of the medial boarder of the patella and were arranged parallel to the muscle fibers. The inferior VL electrode was placed half the distance from the anterior superior iliac spine (ASIS) and the lateral boarder of the patella with the superior electrode placed 1 cm distal to the VL motor point. To accurately locate the VL motor point, the superior portion of the VL was probed with a low‐intensity electrical stimulus. The location that produced the greatest VL muscle twitch was selected for the electrode placement. A ground electrode (10 mm diameter) was applied to the left patella. EMG signals were recorded using ML138BioAmp (common mode rejection ratio >85 dB at 50 Hz, input impedance 200 MΩ) with a 16‐bit analog‐to‐digital conversion, sampled at 4000 Hz (ADInstruments, Sydney, Australia). Fourth‐order Bessel filter was used to filter the raw EMG signals between 20 and 500 Hz.

The quadriceps were stimulated during all maximal isometric knee extensor trials by supramaximal doublets applied to the femoral nerve (200‐*μ*sec square pulse) at 100 Hz and 10 Hz by two high voltage (400 V) constant current stimulators connected in series (Digitimer DS7AH; Digitimer, Hertfordshire, UK). Previous research observed that the stimulation frequency–force response shifted to the right and left with higher and lower *T*_m_, respectively, suggesting that different stimulation frequencies are needed to probe muscle contractile responses to manipulation of *T*_m_ (De Ruiter et al. 1999).

A 20‐mm‐diameter surface electrode was positioned over the nerve in the femoral triangle for cathodal stimulation. The location of the cathode was identified at rest by moving a ball probe and applying low‐intensity stimulation (30 mA) to premarked sites within the femoral triangle. The area that produced the highest evoked compound muscle action potential (M‐wave) in both VL and VM and knee extensor twitch torque was chosen for cathode placement. The anode was custom made using a 5 × 9 cm section of aluminum foil covered with conducting gel that was placed midway between the greater trochanter and the iliac crest. Stimulation intensity to be used during testing for each method of stimulation was determined by progressively increasing the current in 10 mA increments until plateaus occurred in twitch amplitude and M‐waves. Supramaximal stimulation was ensured by increasing the final intensity by 30% (average intensity for 100 Hz doublet, 139 + 25 mA; 10 Hz doublet 144 + 37 mA).

**Figure 1:**
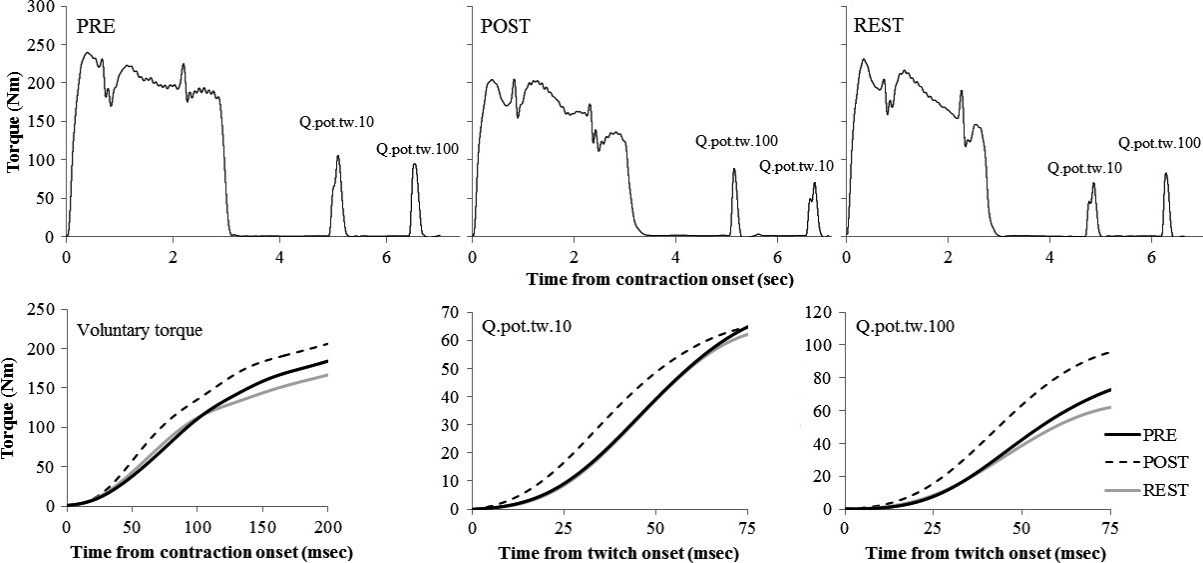
Representative data from a single participant showing the maximal torque trials with interpolated twitches applied during and after the contractions, measured PRE and POST the active warm‐up, and after the REST period. Also shown in the bottom panels are changes in voluntary and evoked torque profiles during the early time periods post contraction onset.

During each maximal trial, two superimposed doublets (100 Hz and 10 Hz) were applied to the femoral nerve when torque had reached a visible plateau. A 1.5 sec time period was used between applied doublets to trigger the second stimulator (Fig. 1). Quadriceps resting potentiated twitches (Q_.pot.tw.100_; Q_.pot.tw.10_) were then produced by delivering two doublets (10 Hz and 100 Hz) to the resting muscle, with the first stimulation in the doublet sequence delivered 2‐ to 3‐ sec post contraction. Doublets were applied in a random order between measurement time‐points (Fig. 1; PRE, POST, REST). There was no difference in measured dependent variables from the order of doublets delivered during contraction.

### Data analysis

Contraction onset (for all torque and EMG signals) was identified using a computer‐based integrated profile method (MATLAB, The Mathworks, Natick, MA), which compares a time normalized function to a normalized function of the integrated signal (Allison et al. 2008; Siegler et al. 2014). The point in time where the two normalized functions are the greatest distance apart is identified as signal onset, as this point indicates where the signal of interest begins to rapidly increase with respect to time.

Torque recordings were used to analyze, (1) the maximal voluntary torque recorded during the contraction excluding the point of stimulation (MVT, Nm); (2) rate of torque development (RTD) was calculated as the average slope of the torque‐time curve (Δtorque/Δtime) during the time periods 0–25 msec, 0–50 msec, 0–75 msec and 0–100 msec post contraction onset; and (3) maximum RTD (RTDmax) was calculated as the greatest average 10 msec slope throughout the first 100 msec of the contraction. Voluntary RTD variables were normalized to MVT for analysis.

Voluntary activation (VA) was estimated from the 10 Hz and 100 Hz stimulations, respectively, using the superimposed twitch technique (Merton 1954) according to the following formula (Strojnik and Komi 1998): VA (%) = 100 – [*D* * (*T*_sup_/MVT)/Q_.pot.tw.max_] * 100, where *D* is the difference between the torque level just before the superimposed twitch (*T*_sup_) and the maximum torque recorded during the twitch, MVT is maximal voluntary torque during the entire contraction (not including the twitch response), and Q_.pot.tw.max_ is the maximal amplitude of the resting potentiated twitch for either the 10 Hz or 100 Hz doublet. Therefore, VA was calculated for both stimulation techniques (VA_10_, VA_100_).

In addition to the maximal amplitude, the following variables were calculated from Q_.pot.tw.10_ and Q_.pot.tw.100_: (1) the time to peak twitch (TPT); (2) the ½ relaxation time (½ RT), calculated as the time from the peak amplitude until 50% of the maximal amplitude had been reached; (3) average slope of the torque‐time curve during the time periods 0–25 msec, 0–50 msec, and 0–75 msec post onset of the twitch; and (4) maximal rate of twitch development based on the greatest average 10 msec slope throughout the twitch. Slope of the torque‐time curves were only calculated up to 75 msec because TPT was always reached 75–100 msec post twitch onset for the 100 Hz doublet. The rate derived measures from the twitches were normalized to the respective maximal twitch amplitudes for data analysis. The low‐ to high‐frequency torque ratio was calculated (10/100 Hz) from the Q_.pot.tw.max_ measured from each doublet.

All EMG variables during maximal contractions were normalized to the respective M‐waves elicited during each contraction for data analysis (EMG/M, %). EMG recordings were used to analyze the following variables from each MVT; (1) the electrically evoked muscle compound action potential (M‐wave) from the first response to the 10 Hz doublet, calculated from the peak‐to‐peak amplitude of the VL and VM EMG raw signal elicited during contraction; (2) the maximal amplitude of the VL and VM EMG signal during MVTs based on processing the greatest average 250 msec root‐mean‐square (RMS) value; (3) the rate of EMG rise for VL and VM calculated from the average slope of the RMS EMG‐time curve during the time periods 0–25 msec, 0–50 msec, 0–75 msec and 0–100 msec post contraction onset.

Skin temperature (*T*_S_) was not different between the right and left legs, therefore data were averaged between the legs for analysis.

### Reliability

Based on the four preexercise maximal trials at the start of each experimental session (Table 2), mean ± SD within‐day within‐subject coefficients of variation (%) for MVT were 4.2 ± 1.2 (range 2.6–7.0), VA_10_ 3.5 ± 1.8 (range 1.5–9.3), VA_100_ 5.3 ± 3.8 (range 0.8–11.7), Q_.pot.tw.max.10_ 3.7 ± 2.6 (range 0.7–10.3), and Q_.pot.tw.max.100_ 3.3 ± 2.9 (range 0.4–14.3). Mean between‐day within‐subject coefficients of variation for MVT were 5.5 ± 1.9 (range 3.7–8.7), VA_10_ 3.5 ± 1.6 (range 1.7–7.7), VA_100_ 5.9 ± 3.3 (range 1.2–11.6), Q_.pot.tw.max.10_ 5.3 ± 2.2 (range 1.7–7.7), and Q_.pot.tw.max.100_ 5.1 ± 3.0 (range 2.0–11.3).

**Table 2:** Preexercise muscle function data for the experimental conditions in this study

	Heat	Control
MVT (Nm)	232.5 ± 49.8	235.0 ± 47.3
Q_.pot.tw.max.10_ (Nm)	107.5 ± 12.7	105.0 ± 10.5
Q_.pot.tw.max.100_ (Nm)	100.1 ± 10.1	95.4 ± 10.7
10/100 Hz	1.07 ± 0.07	1.10 ± 0.09
VA_10_ (%)	91.1 ± 5.1	89.7 ± 5.5
VA_100_ (%)	80.6 ± 14.4	80.7 ± 12.5

MVT, maximal voluntary torque; Q_.pot.tw.max.10_, quadriceps potentiated twitch maximum at 10 Hz; Q_.pot.tw.max.100_, quadriceps potentiated twitch maximum at 100 Hz; VA, voluntary activation.

### Statistical analyses

For PRE exercise values, data from the four maximal contractions and subsequent twitches were averaged to best represent each participant's neuromuscular performance. All data were normally distributed, as assessed from Kolmogorov–Smirnov normality testing. Repeated‐measures analysis of variance (ANOVA) procedures were used to examine changes in dependent variables between each protocol (Heat, Con) over time (PRE, POST, REST). Mauchly's test was used to assess for sphericity and in any cases of violation the Greenhouse–Geisser epsilon correction was used to adjust the degrees of freedom. When a significant f‐value was observed in the ANOVA, post hoc tests with Bonferonni's correction were used to identify differences. Unless otherwise stated data are mean ± SD. Statistical significance was defined as *P* ≤ 0.05.

## Results

### Muscle and skin temperature

**Figure 2:**
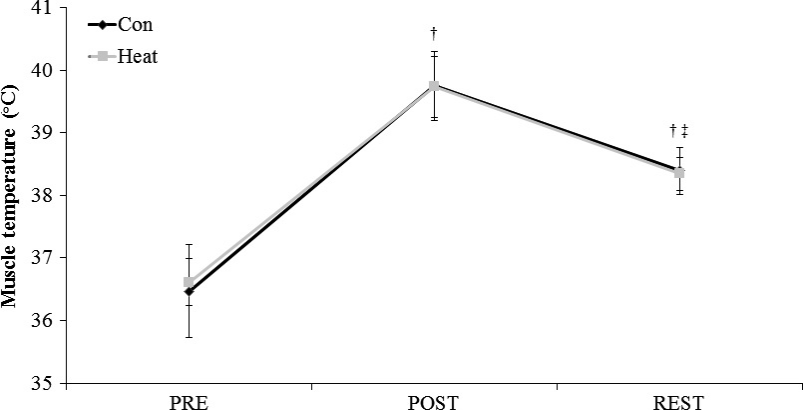
Muscle temperature (*T*_m_) measured before (PRE) and after (POST) the active warm‐up, as well as after the passive rest period for the two experimental conditions (Heat, application of passive heating garment to legs during rest period; Con, control condition). Data are mean ± SD. † is *P* < 0.001 from PRE, ‡ is *P* < 0.001 from POST.

Preexercise *T*_m_ was not different between protocols (CON, 36.5 ± 0.7°C; HEAT, 36.6 ± 0.4°C). No interaction between condition and time was observed for analysis of *T*_m_. A significant time effect was observed with *T*_m_ increased from PRE to POST warm‐up by 3.2 ± 0.7°C (Fig. 2; *P* < 0.001). *T*_m_ was subsequently reduced by 1.4 ± 0.4°C after the REST period (*P* < 0.001), but was still higher than PRE values by 1.8 ± 0.5°C (*P* < 0.001).

*T*_S_ was not different between protocols based on the average values recorded during the first minute of the post warm‐up rest period (CON, 31.3 ± 0.8°C; HEAT, 32.0 ± 3.3°C). Time (*P* < 0.001) and interaction effects were observed for increased *T*_S_ during the rest period with *T*_S_ increased by 1.2 ± 0.5°C during CON, but a greater increase in 4.3 ± 1.8°C observed during HEAT (*P* < 0.001).

### Maximal torque and rate of torque development

**Figure 3:**
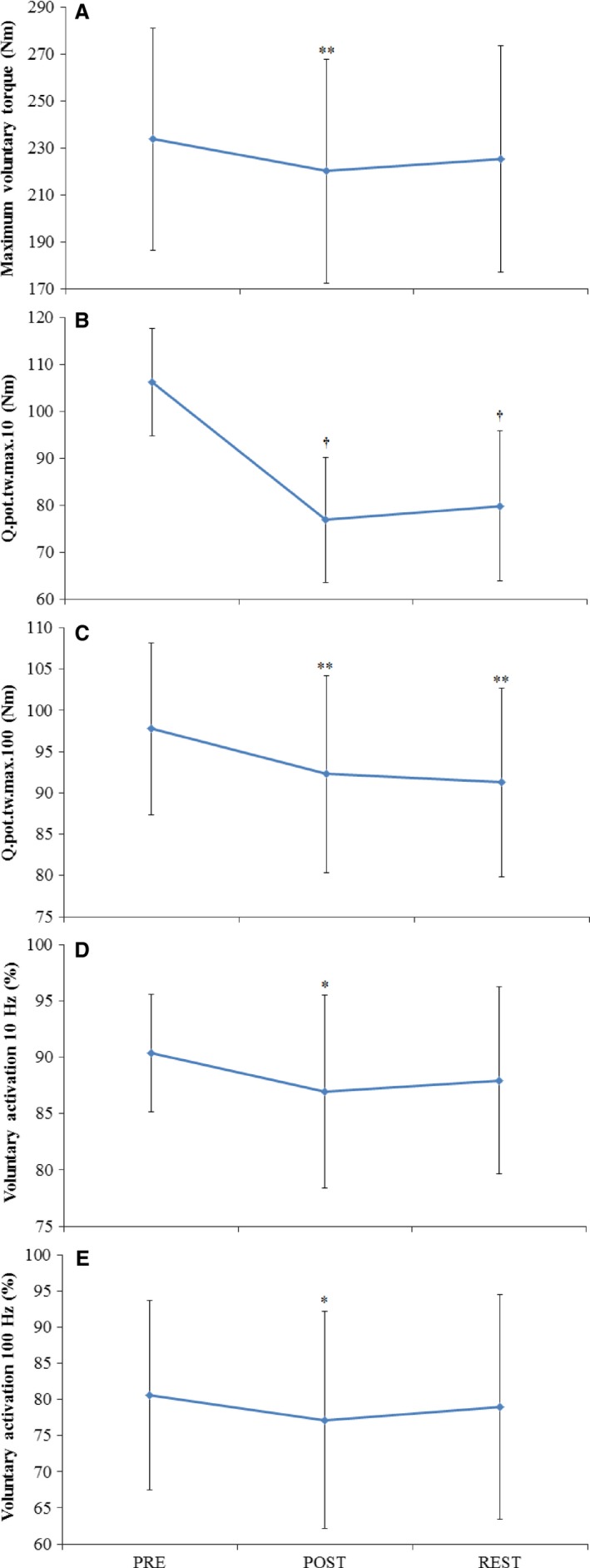
Maximal voluntary torque (A), maximal quadriceps twitch amplitudes (Q_.pot.tw.max_; B, C), and voluntary activation (D, E) measured PRE and POST warm‐up, and after the REST period. Data are mean ± SD for both conditions (Heat and Con) because no interaction effects were observed. * is *P* < 0.05, ** is *P* < 0.01, and † is *P* < 0.001 from PRE.

**Figure 4:**
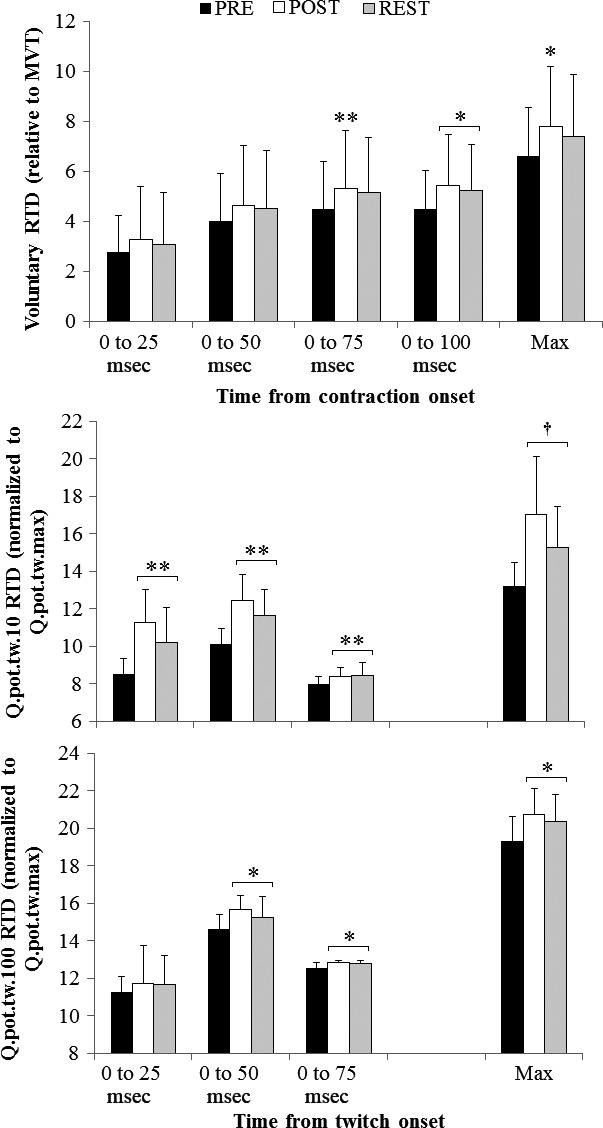
Voluntary rate of torque development (RTD; normalized to maximal torque), and rate of rise in the Q_.pot.tw.10_ and Q_.pot.tw.100_ (absolute slope normalized to Q_.pot.tw.max_ for each doublet) in the indicated time intervals from contraction and twitch onset, measured PRE and POST warm‐up, and after the REST period. Data are mean and SD for both conditions (Heat and Con) because no interaction effects were observed. * is *P* < 0.05, ** is *P* < 0.01, and † is *P* < 0.001 from PRE.

MVT was reduced from PRE to POST warm‐up by 5.6 ± 7.0% (Figs. 1, 3; *P* = 0.006). MVT after the REST period was not different from PRE or POST warm‐up values. RTD in time intervals of 0–25 and 0–50 msec post contraction onset did not change during the experiment (Figs. 1, 4). Significant time effects were only observed for RTD max (*P* = 0.005), and RTD in time intervals of 0–75 (*P* = 0.050) and 0–100 msec (*P* = 0.006) post contraction onset. RTD max and RTD 0–75 msec were increased from PRE warm‐up values by 19.8 ± 33.3% (*P* = 0.012) and 19.4 ± 27.5% (*P* = 0.002), respectively, at the POST warm‐up measurement only. RTD 0–100 msec was increased from PRE warm‐up values by 21.9 ± 31.5% (*P* = 0.003) at POST warm‐up, and remained elevated from PRE values after the REST period by 18.0 ± 34.1% (*P* = 0.045).

### Evoked contractile properties

Vastus lateralis (VL) and vastus medialis (VM) M‐waves did not change during the experiment (Table 3). Q_.pot.tw.max.10_ was reduced from PRE to POST warm‐up by 27.6 ± 10.2% (Figs. 1, 3; *P* < 0.001), and values remained lower after the REST period (*P* < 0.001). The rate of rise of Q_.pot.tw.10_ was increased from PRE to POST warm‐up in time intervals of 0–25, 0–50, and 0–75 msec post contraction onset between 5.2% and 32.2% (Fig. 4; *P* < 0.001), and remained higher than PRE after the REST period (*P* < 0.01). The maximum rate of rise of Q_.pot.tw.10_ was increased from PRE to POST warm‐up by 28.5 ± 16.7% (*P* < 0.001), and remained higher after the REST period (*P* < 0.001). TPT of the Q_.pot.tw.10_ did not change during the experiment (Table 4). ½ RT of the Q_.pot.tw.10_ was reduced from PRE to POST warm‐up by 25.9 ± 8.8% (*P* < 0.001), and remained lower after the REST period (*P* < 0.001).

**Table 3:** M‐waves, maximal amplitude of vastus lateralis (VL) and vastus medialis (VM) electromyograms (EMG) normalized to M‐waves (EMG/M), and rate of rise of the VL and VM muscle signals (EMG/M, % s^−1^) recorded PRE and POST warm‐up, and after the REST period

	PRE	POST	REST
VL M‐wave (mV)	14.0 ± 4.6	13.4 ± 5.3	13.3 ± 5.0
VL EMG/M (%)	8.1 ± 3.5	8.5 ± 5.6	8.8 ± 4.6
VL Rate of rise (% s^−1^)			
0–25 msec	53.2 ± 44.8	58.8 ± 44.6	50.6 ± 28.5
0–50 msec	50.5 ± 30.5	61.4 ± 55.5	69.7 ± 66.1
0–75 msec	38.8 ± 20.1	49.3 ± 44.4	52.3 ± 47.5
0–100 msec	29.7 ± 15.5	35.4 ± 27.8	41.9 ± 29.6
Max	143.8 ± 70.8	146.6 ± 77.8	137.5 ± 45.6
VM M‐wave (mV)	16.9 ± 5.7	16.2 ± 5.2	16.1 ± 4.8
VM EMG/M (%)	6.7 ± 2.2	5.9 ± 1.9	6.4 ± 2.0
VM Rate of rise (% s^−1^)			
0–25 msec	55.5 ± 30.9	52.5 ± 41.4	61.6 ± 36.6
0–50 msec	50.0 ± 22.3	51.4 ± 30.9	56.5 ± 29.4
0–75 msec	35.3 ± 14.7	38.2 ± 18.8	39.5 ± 19.4
0–100 msec	27.6 ± 15.0	28.1 ± 11.8	30.7 ± 17.3
Max	133.2 ± 37.6	134.5 ± 66.9	148.6 ± 61.0

Data are mean ± SD for both conditions as no interaction or condition effects were observed.

**Table 4:** Time to peak twitch (TPT, msec) and ½ relaxation time (1/2 RT, msec) for Q_.pot.tw.10_ and Q_.pot.tw.100_ measured PRE and POST warm‐up, and after the REST period

	PRE	POST	REST
Q_.pot.tw.10_			
TPT (msec)	156.2 ± 5.8	148.2 ± 14.2	149.4 ± 14.7
½ RT (msec)	68.4 ± 9.1	50.8 ± 9.7**†**	52.7 ± 9.5†
Q_.pot.tw.100_			
TPT (msec)	91.4 ± 8.2	76.1 ± 6.9**†**	77.7 ± 6.7†
½ RT (msec)	77.9 ± 16.1	63.0 ± 8.2**	64.5 ± 8.5**

Data are mean ± SD averaged for both conditions, as no interaction or condition effects were observed.

**P < 0.01, and ^†^ *P* < 0.001 from PRE.

Q_.pot.tw.max.100_ was reduced from PRE to POST warm‐up by 5.6 ± 6.9% (Fig. 4; *P* = 0.006), and remained lower than PRE after the REST period (*P* = 0.009). Time effects were observed for the rate of rise of Q_.pot.tw.100_ in time intervals of 0–50 and 0–75 msec post contraction onset, in addition to the maximal rate of rise of Q_.pot.tw.100_ (Fig. 4; p < 0.05). Values were increased from PRE warm‐up between 2.4% and 7.4% POST warm‐up, and remained higher after the REST period (*P* < 0.05). TPT and ½ RT of the Q_.pot.tw.100_ were both shortened POST warm‐up by 16.5 ± 7.2% (*P* < 0.001) and 16.4 ± 16.4% (*P* = 0.002), respectively (Table 3). Values remained lower than PRE after the REST period (*P* < 0.01).

The low‐ to high‐frequency torque ratio (10 Hz/100 Hz for Q_.pot.tw.max_) was reduced from PRE warm‐up values of 1.09 ± 0.08 to 0.84 ± 0.13 POST warm‐up (*P* < 0.001). Values remained reduced after the REST period (*P* < 0.001).

### Central motor output

Significant time effects were observed for VA_10_ and VA_100_ (Fig. 3; *P* < 0.05), with values reduced from PRE to POST warm‐up by 3.5 ± 5.4% and 3.5 ± 4.5%, respectively. Values after the REST period were not different from PRE and POST warm‐up. VL and VM maximal EMG/M, and the rate of rise in VL and VM EMG/M values did not change during the experiment (Table 3).

## Discussion

The aim of this study was to examine the relationship between the manipulation of *T*_m_, voluntary rate of torque development, in vivo measures of muscle contractile function, and central motor output around an active warm‐up and subsequent rest period. Moreover, we examined whether the use of a passive heating garment applied during the post warm‐up rest period. We found some evidence to support our first hypothesis that the active warm‐up would elicit concomitant increases in muscle temperature and voluntary rate of torque development, likely explained by the increased rate‐dependent measures of in vivo muscle contractile function. However, no evidence was found to suggest increased central motor output following the warm‐up. Indeed, an unexpected finding was that the warm‐up induced both central and peripheral muscle fatigue, manifest from declines in voluntary activation and low‐frequency muscle fatigue. As hypothesized, the use of a passive heating garment for the 12.5‐min rest period post warm‐up was insufficient to attenuate declines in muscle temperature. However, rate‐dependent measures of in vivo muscle contractile function remained elevated from pre warm‐up values after the rest period despite declines in muscle temperature.

The results of this study support and extend previous research regarding the use of active warm‐ups prior to sport. This was the first study to show that increased voluntary rate of torque development following an active warm‐up that raised muscle temperature by 3.2 ± 0.7°C was mediated by elevations in rate‐dependent measures of electrically evoked muscle contractile function. These findings extend the work of De Ruiter and colleagues (De Ruiter et al. 1999), who were the first to report increased electrically evoked rate‐dependent measures of muscle contractile function in humans following elevation of muscle temperature. While incremental increases in maximal rate of force development and reductions in ½ relaxation time were observed between temperatures of 22, 25, 31, and 37°C (De Ruiter et al. 1999), it was unclear whether or not temperature manipulation within the physiological range typically observed in the vastus lateralis muscle of humans around active warm‐ups (35–39°C) would induce similar changes. Subsequent papers observed faster in vivo quadriceps contractile responses after warm‐ups but did not provide measurement of *T*_m_ to substantiate this mechanistic relationship (Skof and Strojnik 2007; Girard et al. 2009). After the warm‐up in this study, we found increased rate of torque development in the 10 Hz and 100 Hz twitches in addition to reduced ½ relaxation times. With no change in the early rate of rise in muscle activation, it appears that the increased voluntary rate of torque development was best explained by changes in the intrinsic contractile properties of muscle. It is thought that the increased rate of torque development in muscle following temperature elevation is best explained by the increased ATP hydrolysis and the accelerated attachment of M.ADP.P_i_ to actin (Offer and Ranatunga 2015). A surprising observation in this study was that the changes in intrinsic contractile properties associated with increased muscle temperature appear to have offset manifestations of central and peripheral fatigue following the active warm‐up.

A 5.6% decline in maximal voluntary torque was observed after the warm‐up, which seems explained by both central and peripheral fatigue. Central fatigue was indicated from reductions in voluntary activation, manifest from both the 10 Hz and 100 Hz doublets interpolated upon maximal contractions. The potentially deleterious contribution of central fatigue to voluntary rates of torque development did not manifest through any reductions in early rate of rise in the quadriceps electromyograms (Buckthorpe et al. 2014; Marshall et al. 2014). Therefore, any central fatigue induced from a similar active warm‐up is only likely to impede maximal torque, and not the more important functional parameter for power production of rate of torque development. Peripheral fatigue after the active warm‐up was indicated from declines in the maximal amplitude of both the 10 Hz and 100 Hz resting potentiated twitches, although these declines were more pronounced for the 10 Hz twitch indicating low‐frequency muscle fatigue (Allen et al. 2008). Low‐frequency muscle fatigue, thought to indicate impaired rate of calcium release from the sarcoplasmic reticulum and subsequent binding to the thin myofilaments (Allen et al. 2008), appears to be more pronounced within the physiological range of muscle temperatures examined in this study (De Ruiter et al. 1999). De Ruiter (De Ruiter et al. 1999) examined changes in the rate of force development measured from different stimulation responses during electrically evoked muscle fatigue of the adductor pollicis at the previously described temperatures. At 37°C, the effect of fatigue was to cause a rightwards shift in the force‐frequency relationship (De Ruiter et al. 1999), indicating that muscular fatigue within the physiological range measured in this study (36–39°C) was more likely to manifest as declines in the amplitude of the 10 Hz twitch. In this study, it appears that the extent of low‐frequency muscle fatigue was countered by the temperature‐dependent up‐regulation of intrinsic contractile processes, manifest as increased rate of torque development of both the 10 Hz and 100 Hz twitches, leading to increased voluntary rate of torque development.

The manifestation of peripheral and central fatigue after the warm‐up contrasts two previous reports for no peripheral fatigue and increased central motor output (Skof and Strojnik 2007; Girard et al. 2009). The potential explanations for between study differences can be linked to the different stimulation methods used, and the duration of the warm‐up protocols. In this paper, we used both a 10 Hz and 100 Hz doublet because De Ruiter showed that the stimulation frequency–force response shifted to the right and left with higher and lower *T*_m_, respectively, suggesting that the different stimulation frequencies are needed to accurately probe muscle contractile responses (De Ruiter et al. 1999). In contrast, Skof used single twitches to explore muscle contractile parameters and a 800 msec pulse train to measure central activation (Skof and Strojnik 2007), while Girard used a 100 Hz doublet stimulation to quantify both muscle contractile function and voluntary activation (Girard et al. 2009). Thus, our 10 Hz stimulation is best matched to the single pulse used by the former, whereas we used the same 100 Hz doublet stimuli as the latter. Based on comparable methods, our findings for the 10 Hz stimulation method contrast the potentiation of the single twitch reported by Skof (Skof and Strojnik 2007), but are similar for the reduction in 100 Hz doublet amplitude reported by Girard (Girard et al. 2009) following a strength but not running‐based warm‐up. Therefore, the primary difference with regard to twitch responses is the manifestation of low‐frequency muscle fatigue. The peripheral and central fatigue in this study is probably explained by the overall density of the warm‐up used in this study. The total duration for the warm‐up protocols outlined by Skof (Skof and Strojnik 2007) and Girard (Girard et al. 2009), which utilized running and sprinting tasks similar to this study, were 25–27 and 40–43 min, respectively. In contrast, the duration of the warm‐up in this study, primarily based on parts 1 and 3 of the FIFA‐11, was ~17.5 min and approximately used the same type and amounts of high‐velocity movements, thus increasing the density of the warm‐up. Whether extending the duration of the warm‐up to offset the peripheral and central fatigue is more efficacious for clinical practice is unclear. It must be noted that only maximal torque output and voluntary activation at this point of the contraction was lowered after the warm‐up. Both variables returned to pre warm‐up levels after the rest period, therefore any negative clinical consequence of the fatigue‐induced after the warm‐up used in this study appears negligible.

As hypothesized, the application of a passive heating garment for the 12.5‐min rest period following the active warm‐up was insufficient for attenuating declines in muscle temperature, measured at a depth of 4 cm. Moreover, any temperature effect on muscle fibers closer to the skin, not measured in this study, did not manifest as a significant effect on both intrinsic contractile properties of muscle or voluntary rate of torque production. Previous research found that the passive heating garment needed to be applied for at least 28 min for muscle temperature declines at a depth of 2 cm to be attenuated (Faulkner et al. 2013b). In the context of soccer, the application of a passive heating garment may have better application for reserve players on the sideline who are waiting to play and are not otherwise continuously active. These players often perform active rewarm‐ups of 3–5 min duration which can increase muscle temperature approximately 1°C (Lovell et al. 2013). Thus, in combination with the application of a passive heating garment for periods of time >20 min, higher muscle temperatures and therefore explosive performance may be better maintained for reserve players who need to be consistently ready to enter match play. Future research is required to explore this context of maintaining physiological performance capacity in reserve players.

As expected, we observed a decline in muscle temperature measured at a 4 cm depth of 1.4°C after the 12.5‐min rest period. This decline is commensurate with trends observed in vastus lateralis muscle temperature data observed in rest periods following a cycling warm‐up (Faulkner et al. 2013a,b), and a 15‐min half‐time rest period during a simulated soccer match (Lovell et al. 2013). In contrast to our hypothesis, voluntary rate of torque development in the time period up to 100 msec post contraction onset, in addition to rate‐dependent measures of muscle contractile function did not decline to pre warm‐up levels. This may be explained by the finding that muscle temperature, while reduced from the end of the warm‐up, was still higher than pre warm‐up levels by 1.8°C. Thus, the beneficial effects of the warm‐up used in this study for enhanced muscle function were maintained, despite the fatigue measured after the warm‐up and the subsequent 12.5‐min rest period.

Some limitations to this study must be mentioned. First, we used an isometric testing model that may not be valid for inferences about voluntary rate of torque development during dynamic concentric and eccentric muscle actions typically performed by soccer players during training and match‐play. Moreover, our measurement model was restricted to an isolated knee extension movement to provide controlled measurement of quadriceps contractile function. Thus, the specificity of findings here with regard to an isometric knee extension model may not be generalizable to lower limb compound movements that involve many lower limb muscles (i.e., sprinting, jumping). However, it has been observed that the rate of torque development from quadriceps twitch responses was positively associated with countermovement squat jump height in elite male volleyball athletes (De Ruiter et al. 2007). Thus, our findings may have relevance for dynamic tasks requiring rapid torque output from the knee extensors. Indeed, it has been reported that performance variables such as 20‐m sprint time and vertical jump are increased in amateur soccer players following a warm‐up similar to that used in this study, suggesting that enhanced quadriceps contractile function observed in this study may contribute to improved post warm‐up dynamic performance in soccer players (Bizzini et al. 2013). Our study was conducted in a controlled laboratory environment, therefore, we cannot generalize our outcomes into the range of environmental conditions experienced by players in competition. Finally, we used a modified version of the FIFA‐11+ warm‐up. We cannot be sure that inclusion of part 2 including various lower limb strength and conditioning exercises would interfere with the positive contractile performance outcomes observed here.

In summary, we observed that an active warm‐up elevated muscle temperature by 3.2°C and was associated with concomitant increases of between 20% and 30% in voluntary rate of torque development in the early time intervals after contraction onset. These increases in voluntary rate of torque development appear explained by elevations in rate‐ dependent measures of intrinsic muscle contractile function. Application of a passive heating garment did not attenuate declines in muscle temperature during a 12.5‐min rest period. However, the 12.5‐min rest period does not appear long enough to reduce muscle temperature back to preexercise levels, therefore muscle contractile function and voluntary rate of force development remained higher than preexercise.

## Conflict of Interest

None declared.
